# Not All Home Care is Created Equal: The Relationship Between Sources of Care and Recipients’ Perceived Control

**DOI:** 10.1093/geroni/igaa057.3232

**Published:** 2020-12-16

**Authors:** Molly Wylie

**Affiliations:** University of Massachusetts Boston, Boston, Massachusetts, United States

## Abstract

Perceived control is an important psychological resource for middle-aged and older adults. Aging in place may help foster feelings of control and autonomy, yet many community-dwelling older adults must rely on others for physical assistance. Little is known about psychological reactions to receiving this support. This study investigated how receiving home care from different sources was associated with two facets of perceived control (mastery and perceived constraints) among adults with varying levels of physical disability. Data was drawn from the 2012 and 2014 waves of the Health and Retirement Study. Community-dwelling adults aged 50 years and older receiving help for at least one activity of daily living (ADL) impairment (N = 884) reported their relationship to each respective caregiver (formal professional and/or informal family or friend), level of ADL impairment, and ratings of perceived control. Ordinary least squares (OLS) regression was used to examine the association between type of support and perceived control and the moderating effect of physical disability on that relationship. Receiving formal or a combination of formal and informal support was related to perceptions of greater control over one’s life, but only in terms of mastery. The level of one’s ADL impairment did not have a moderating effect on the relationship between support type and perceived control. Findings suggested that the type of instrumental support adults receive in their home has implications for specific facets of perceived control. Those receiving formal or mixed support had perceptions of greater mastery than those only receiving informal support.

